# Context Modulates Congruency Effects in Selective Attention to Social Cues

**DOI:** 10.3389/fpsyg.2018.00940

**Published:** 2018-06-12

**Authors:** Andrea Ravagli, Francesco Marini, Barbara F. M. Marino, Paola Ricciardelli

**Affiliations:** ^1^Department of Psychology, University of Milano-Bicocca, Milan, Italy; ^2^Department of Psychology, University of Nevada, Reno, Reno, NV, United States; ^3^Swartz Center for Computational Neuroscience, Institute for Neural Computation, University of California, San Diego, San Diego, CA, United States; ^4^Milan Center for Neuroscience, Milan, Italy

**Keywords:** gaze discrimination, head orientation, social cues, social attention, distraction context manipulation paradigm, proactive control, conflict adaptation, proportion congruency effect

## Abstract

Head and gaze directions are used during social interactions as essential cues to infer where someone attends. When head and gaze are oriented toward opposite directions, we need to extract socially meaningful information despite stimulus conflict. Recently, a cognitive and neural mechanism for filtering-out conflicting stimuli has been identified while performing non-social attention tasks. This mechanism is engaged proactively when conflict is anticipated in a high proportion of trials and reactively when conflict occurs infrequently. Here, we investigated whether a similar mechanism is at play for limiting distraction from conflicting social cues during gaze or head direction discrimination tasks in contexts with different probabilities of conflict. Results showed that, for the gaze direction task only (Experiment 1), inverse efficiency (IE) scores for distractor-absent trials (i.e., faces with averted gaze and centrally oriented head) were larger (indicating worse performance) when these trials were intermixed with congruent/incongruent distractor-present trials (i.e., faces with averted gaze and tilted head in the same/opposite direction) relative to when the same distractor-absent trials were shown in isolation. Moreover, on distractor-present trials, IE scores for congruent (vs. incongruent) head-gaze pairs in blocks with rare conflict were larger than in blocks with frequent conflict, suggesting that adaptation to conflict was more efficient than adaptation to infrequent events. However, when the task required discrimination of head orientation while ignoring gaze direction, performance was not impacted by both block-level and current trial congruency (Experiment 2), unless the cognitive load of the task was increased by adding a concurrent task (Experiment 3). Overall, our study demonstrates that during attention to social cues proactive cognitive control mechanisms are modulated by the expectation of conflicting stimulus information at both the block- and trial-sequence level, and by the type of task and cognitive load. This helps to clarify the inherent differences in the distracting potential of head and gaze cues during speeded social attention tasks.

## Introduction

Head and gaze directions are the most important pieces of information used by human perceptual-cognitive systems during social interactions to determine where another person is attending (Argyle and Cook, 1976; Emery, 2000). Therefore, characterizing how head and eye cues are combined during perceptual and cognitive processing – particularly when these cues are conflicting (e.g., Balsdon and Clifford, 2017) – is fundamental in understanding human behavior in the context of social interactions. When we observe other people’s faces, it is common that head and gaze directions are not aligned (e.g., right-oriented head with left-averted gaze). When one needs to determine where another person is attending based on conflicting directional information delivered by head and gaze, this conflict must be resolved by perceptual-cognitive systems (e.g., Perrett et al., 1992) – however, the mechanisms underlying head-gaze conflict resolution are not fully understood yet (e.g., Langton, 2000; Moors et al., 2016; Otsuka et al., 2016a,b).

It is well-known that head-gaze conflict might lead to biases in the perceived gaze direction during tasks requiring the integration of eye and head orientation (e.g., Gibson and Pick, 1963; Cline, 1967; Anstis et al., 1969; Otsuka et al., 2015, 2016a,b; Moors et al., 2016; Balsdon and Clifford, 2017). In a frequently investigated bias known as “repulsive effect” or “overshoot effect,” the perceived gaze direction is slightly biased toward the *opposite* direction relative to the direction in which the head is oriented (e.g., Gibson and Pick, 1963; Anstis et al., 1969; Masame, 1990; Gamer and Hecht, 2007; but for a different bias^1^, see also Cline, 1967; Maruyama and Endo, 1983; Langton et al., 2004). The overshoot effect and similar biases occurring in the presence of conflictual head-gaze cues help to characterize the mechanisms of integration of head and gaze information. One possibility is that these biases derive from an imbalance in the weights attributed to directional information from the head and the eyes, respectively, during the integration of multiple and conflicting directional cues. For example, if the head of an observed face has a rightward tilt and the eyes are centered, an excessive negative weight attributed to the directional cues from the head might result in the gaze being perceived as directed slightly to the left – thus resulting in the overshoot effect. However, multiple accounts exist regarding the integration of head and eye information during face perception (e.g., Langton, 2000; Ricciardelli and Driver, 2008; Nummenmaa and Calder, 2009; Otsuka et al., 2014).

A frequently used taxonomy distinguishes between global and local information conveyed by face stimuli. Global information corresponds to the overall form (thus including head orientation) while local information corresponds to finer-grain details (thus including gaze direction). The overshoot effect and other perceptual modulations on perceived gaze direction driven by head orientation may depend on how local and global perceptual cues are combined to form a coherent percept (e.g., Tanaka and Farah, 2007; McKone, 2008; Tanaka and Simonyi, 2016). Importantly, the distinction between local and global cues reflects perceptual processes with different temporal dynamics: for example, monkey electrophysiology research has demonstrated that global and local information are processed with peculiar spatio-temporal dynamics (De Souza et al., 2005; Rolls, 2007). Although this finding seems to suggest a relative reciprocal independence of head and eye cues during perceptual face processing, whether global and local cues are processed in parallel or are integrated is still a matter of debate. Perrett et al. (1992) in an electrophysiological study found that cells in the superior temporal sulcus (STS) have an extensive sensitivity to head views, gaze direction and body postures. Interestingly, the authors argue that the primary function of this sensitivity is to signal the direction of attention of other individuals and that gaze direction is the best cue to indicate the focus of attention. Accordingly, it was reported that the sensitivity to gaze direction, when not in accordance with head orientation or body posture, could override the sensitivity of head view, which in turn could override body posture. The authors, thus, postulate the existence of a direction-of-attention detector (DAD) that combines in a hierarchical manner the information from separate detectors that analyze the direction of the eyes, head and body. This hierarchy in combining eye, head, and body cues is achieved thanks to a network of inhibitory connections. That is, information from the eyes can directly inhibit cells coding an inappropriate head direction, but not vice-versa, and information about a particular head angle can inhibit cells coding a conflicting body position, but not vice versa. In contrast, other studies have suggested that information from head orientation is not completely suppressed when in conflict with gaze direction (Langton, 2000; see Emery, 2000; Langton et al., 2000 for reviews). This implies that head orientation contributes somehow to the computation of attention direction even when the head angle conflicts with the direction of gaze. Ricciardelli and Driver (2008) provided behavioral evidence showing that the mechanisms responsible for processing head and gaze direction show some hierarchical organization and may not operate completely independently (see also Hietanen, 1999, 2002; Bayliss et al., 2004). Accordingly, the overshoot effect also suggests the existence of some degree of integration between local and global cues. However, the reciprocal weights attributed to head and eye cues during this integration are not completely understood yet (e.g., Perrett et al., 1992; Langton, 2000; Ricciardelli and Driver, 2008; Otsuka et al., 2014). Ricciardelli and Driver (2008) proposed that the visual system might attribute different weights to particular head and eye cues according to their visibility and the required speed of the gaze discrimination judgement. However, it is unclear what mechanisms intervene to resolve conflicts between head orientation and gaze direction.

A possibility is that top-down cognitive control processes intervene to resolve this conflict. Is the perceptual decision about where another person is attending susceptible to top-down control? If so, perceptual-cognitive systems might intervene to filter-out one source of information (e.g., eye cues or head cues) from the cue integration process when the observer has prior knowledge that a source of information is irrelevant within a given face-processing context. In the present study, we modeled this scenario using a recently introduced experimental paradigm for the characterization of proactive and reactive cognitive-attentional control mechanisms in the presence of conflicting and distracting information – namely, the Distractor Context Manipulation paradigm (Marini et al., 2013, 2015, 2016). In this paradigm, different levels of expectation for conflicting information are created at the block level by using multiple types of experimental blocks with different probabilities of conflict, in addition to no-conflict trials that are both intermixed with conflict trials (in “Mixed” blocks) and presented in isolation in a separate block (“Pure” block). One type of block had an expectation of distraction because both distractor-absent and distractor-present trials were intermixed (the Mixed block), while the other type of block included only distractor-absent trials (the Pure block) and therefore engendered no expectation for distractors. The comparison of speeded performance (e.g., reaction times or inverse-efficiency scores) in distractor-absent trials of the Mixed blocks vs. distractor-absent trials of the Pure blocks might reveal a behavioral cost in Mixed blocks, which has been related to the recruitment of mechanisms for conflict resolution and distraction-filtering (Marini et al., 2013, 2016) and hence termed “distraction-filtering cost.” Because this distraction-filtering cost was inversely correlated to the behavioral cost caused by conflict on conflict-present trials (i.e., incongruent vs. congruent trials), it is considered that the behavioral signature of the proactive engagement of a distraction-filtering mechanism is invoked in potentially distracting contexts in order to limit the negative impact of conflicting distraction (Marini et al., 2013, 2016). This distraction-filtering mechanism was shown to be sensitive to contextual factors because it was modulated both proactively (for example, in relation to the probability of occurrence of conflicting distractors within a given experimental block) as well as reactively (for example, after the occurrence of conflicting distractors in the immediately preceding trial) (Marini et al., 2013, 2016). This distraction-filtering mechanism has been described in several different paradigms of visual and cross-modal attention (Marini et al., 2013, 2016), and appears to be a general mechanism of cognitive-attentional control. Therefore, it is plausible to hypothesize that a similar mechanism would intervene also in other types of attention tasks – such as social attention tasks. Social attention tasks, such as gaze-direction or head-orientation discrimination tasks, may require selecting task-relevant information while filtering-out irrelevant cues, particularly in the presence of conflicting cue information. However, whether or not a distraction filtering mechanism intervenes during attention tasks with social cues remains to be established.

Here, our general working hypothesis was that a similar cognitive control mechanism for filtering-out conflicting and/or distracting information might be recruited in the context of attention to social cues in order to resolve the potential conflict between head and gaze cues (e.g., as in the overshoot effect). The rationale rests on the fact that in Ricciardelli and Driver’s (2008) study speeded gaze direction judgments were faster when head and gaze are oriented to the same direction (congruent trials) and slower when oriented to opposite directions (incongruent trials). However, head-gaze congruency effect [reaction time (RT) in incongruent minus congruent trials] reversed in the absence of task-imposed speed constraints. Under time pressure, the global head orientation appeared to be weighted more heavily, so that the gaze toward the same side as head deviations then became easier to judge rapidly; however, when the gaze toward the opposite side resulted in the overshoot effect. Intriguingly, this suggests that the reciprocal weights of relevant and irrelevant information can be adjusted depending on the speeded context of the task.

Here, we conducted three experiments in which we adapted the DCM paradigm to both gaze-direction and head-orientation discrimination tasks in contexts with different proportions of trials with congruent/incongruent head orientation and gaze direction, respectively. In Experiment 1, we wanted to investigate if head orientation can be filtered-out during gaze direction discriminations as indicated by the incursion of a distraction-filtering cost. If head-orientation is filtered-out during gaze-direction discrimination tasks, then a distraction-filtering cost should be found on trials with potential distraction compared to trials with no distraction. Moreover, the congruency effect (incongruent minus congruent trials) should be modulated both proactively and reactively by conflict probability. Therefore, we expected to observe larger distraction filtering costs and smaller congruency effects in contexts with high probability of conflicting distraction relative to contexts with low probability of conflicting distraction. Moreover, we expected that the overall proactive distraction-filtering mechanism, whose recruitment corresponds to the magnitude of the distraction-filtering cost, would be enhanced reactively after trials with conflicting distractors. In Experiment 2, we investigated the reverse type of task, in which participants performed head orientation discriminations while gaze direction needed to be filtered (Experiment 2). When the task-irrelevant information was gaze direction, we expected a different pattern of results. Because gaze direction is processed more locally than head orientation (Watanabe et al., 1999) and given the well-known presence of a global advantage in information processing (e.g., Navon, 1977; Mills and Dodd, 2014), it is plausible that the negative impact of conflicting gaze on the head orientation task would be smaller or even absent. If so, neither proactive nor reactive modulations of conflict should emerge in Experiment 2. In Experiment 3, we tested the effects of increasing cognitive load in filtering-out gaze direction during head orientation discriminations. Because cognitive load is thought to modulate the efficiency of distraction-filtering (Marini et al., 2013), we expected that conflict-related effects would emerge with a similar pattern to the one predicted for Experiment 1 when the cognitive load of the head orientation discrimination task was increased.

## Experiment 1

### Materials and Methods

#### Participants

Twenty-two participants took part in Experiment 1 (mean age 22.4, range 18–26, 17 females, 19 right-handed). Two participants were excluded from the analysis due to the high number (more than 48 trials) of omitted responses. All with normal or corrected-to-normal vision and with no known neurological or psychiatric condition. All participants were recruited among Psychology students, gave their written informed consent to take part in the study, and received course credit for their participation. To ensure no waste of time and resources the sample size of all experiments was determined on the basis of previous studies (Marini et al., 2013, 2015, 2016) or on *a priori* power analysis.

#### Ethics Statement

All the experiments were approved by the ethical committee of the University of Milano-Bicocca and were conducted in accordance with the ethical standards laid down in the 1964 Declaration of Helsinki (World Medical Association, 2001) and fulfilled the ethical standard procedure recommended by the Italian Association of Psychology (AIP). All the experimental protocols were also approved by the ethics committee of the University of Milano-Bicocca.

#### Apparatus and Materials

Participants sat in a dimly illuminated room at a distance of 57 cm from the central fixation point of a 21″ computer screen (Samsung SyncMaster 1100p plus, 1280 × 1024 pixel, refresh rate 85 Hz). The experimental paradigm was programmed in Matlab (MathWorks, Inc.) with Psychtoolbox 3.0 (Kleiner et al., 2007). Responses were collected through button presses on a USB keypad.

Stimuli consisted of Caucasian faces with different gaze orientation and different head orientation. Photographs from the Radboud Faces Database (Langner et al., 2010) were modified using Java Psychomorph 6 (Tiddeman et al., 2005) in order to generate an average face for each gender (male/female), gaze orientation (left/right), and head orientation (centered or tilted 45° left/right). This procedure generated a total of 12 unique face stimuli. Additionally, we used a phase-spectrum perturbation technique in Matlab for generating another 12 scrambled faces, which we used as masking stimuli.

#### Procedure

Each trial started with the presentation of a face stimulus (subtending a visual angle of 14.5° vertically by 10° horizontally) on a uniform gray background. Participants were instructed to indicate gaze direction (“target” dimension; left or right) as fast and as accurately as possible, while ignoring head orientation (“distractor” dimension; left, centered, or right). The face stimulus stayed on-screen until either participant’s response or for 1000 ms (whichever occurred first), and was immediately followed by a visual mask (100 ms). The inter-trial interval was jittered between 300 and 600 ms. Three types of trial were used: (1) distractor-absent trials, with no lateral tilt of the head orientation (i.e., the head was straight); (2) congruent distractor trials, with the head orientation tilted in the same direction as the gaze (either both left or both right); and (3) incongruent distractor trials, with the head orientation tilted in the opposite direction as the gaze (either gaze left and head right, or vice-versa).

**FIGURE 1:**
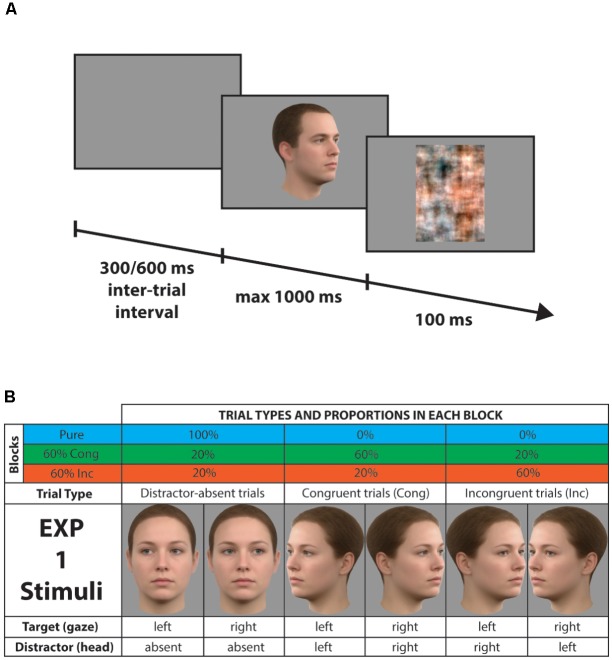
Examples of stimuli, trial types, and procedure (Experiment 1). **(A)** Example of a single trial. After 300 to 600 ms, a face appeared for 1000 ms at most and the participant responded according to the gaze direction. A mask followed the face for 100 ms. **(B)** Table of the proportion of the different trial types in each block. In the lower part of the table, the pictures are the complete female set of stimuli with the description of gaze and head orientation beneath them. The male set is not shown here for brevity, but see **Figure 5**.

In order to investigate the functioning of proactive mechanisms for controlling the conflict emerging when gaze direction was task-irrelevant, we used the same distraction context manipulation, which has been used in previous work for identifying and characterizing mechanisms of distraction filtering (Marini et al., 2013, 2015, 2016). This paradigm typically involves two types of blocks (see **Figure 1**): Pure blocks, in which distractor-absent stimuli are presented on 100% of trials, and Mixed blocks, in which distractor-absent stimuli are presented on 20% of trials and distractor-present stimuli are presented on 80% of trials. Here, we used two different types of Mixed blocks: (i) the 60% Congruent block (60% Cong), consisting of 60% congruent distractor trials, 20% incongruent distractor trials, and 20% distractor-absent trials; (ii) the 60% Incongruent block (60% Inc), consisting of 60% incongruent distractor trials, 20% congruent distractor trials, and 20% distractor-absent trials. Every block was preceded by an on-screen cue that informed participants about the type of upcoming block (Pure, 60% Cong, 60% Inc). Prior to the beginning of the experiment, written instructions and examples of stimuli were shown on the screen and participants performed 30 practice trials. Each experiment consisted of 960 trials divided in 15 blocks (5 blocks of each type, presented in a counterbalanced sequence) and had an average duration of 25 min.

### Results

#### Data Handling

Reaction time and response accuracy were measured. RTs were filtered to eliminate outliers, defined as those trials on which RT was either below 200 ms or above the mean plus three standard deviations computed in log values (Ratcliff, 1993). In order to control for the speed-accuracy tradeoff, inverse efficiency (IE) scores were calculated by dividing RTs by the proportion of accuracy (Townsend and Ashby, 1983). We conducted full statistical analyses on IE scores. We report analyses on IE scores with ms_a_ as the unit of measurement where “a” indicates that the ms value is adjusted (Marini et al., 2015).

We carried out four main analyses, each with a different purpose. In the first one we analyzed the distractor-absent trials in the Pure blocks (baseline) that we compared to the distractor-absent trials in Mixed blocks to estimate the cost of the engagement of the strategic filtering mechanism in the blocks where the distraction was present. By doing this, we expected to measure a behavioral cost (slower responses) in distractor-absent trials of the Mixed blocks (distracting context), compared to the same distractor-absent trials within the Pure blocks (distractor-free context). The second analysis focuses on the sequential effects that could be present in the distractor-absent trials. In Mixed blocks only, we compared the distractor-absent trials that followed a distractor-absent trial (previous distractor-free context) to the distractor-absent trials that followed a distractor-present trial (previous distraction context). If in the first analysis, we found a distraction-cost, and now we do not find a significant difference between the previous distractor-free and previous distraction context, it means that in the Mixed blocks, only a proactive/strategic control is at play. By contrast, if we found a significant difference, then a reactive response strategy has been adopted because of having experienced distraction in the preceding trial. However, in the latter case the problem is to establish whether the behavioral cost found in the first analysis is all or only in part accounted by the reactive control. To this end, we need to compare the distractor-absent trials that followed a distractor-absent trial in the Mixed blocks to the distractor-absent trials that followed a distractor-absent trial in the Pure blocks. If IEs for distractor-absent trials following a distractor-absent trial between Pure and Mixed blocks did not differ, then the cost we measured in Mixed blocks (compared to Pure blocks) in the first analysis would only due to reactive control. In contrast, if the IEs in Pure and Mixed blocks were different, despite the experience of the previous trial was identical (a distractor-absent trial), then we could safely conclude that a proactive/strategic control is also at play in the Mixed blocks and a distractor-expectation cost is present. Therefore, performing the analysis on sequential effects allows us to choose the correct interpretation of the results of our first analysis. The third analysis regards the congruency effects in Mixed blocks. From Ricciardelli and Driver’s (2008) study appears that the congruency effect can be modulated by some contextual factors such as, for example, some temporal constraints implemented in the experimental paradigm used. In our third analysis, we tested a contextual hypothesis. Specifically, we tested whether the frequency of conflict (i.e., high in 60% Incongruent blocks or low in 60% Congruent blocks) could modulate the head-gaze congruency effect because of the different type of the information (i.e., head or gaze) that needs to be processed in the two different conditions of conflict (high vs. low). The fourth and last analysis focuses on the Gratton effect. In previous works with non-social stimuli, it has been reported that the congruency effect is lower after incongruent trials than after congruent ones (Gratton effect). This is taken as an index of a reactive adaptation to conflict and it may well be that, in Mixed blocks, it is modulated by the distraction probability. With this analysis we test whether or not the mechanisms that control conflicting information are the same for social and non-social stimuli (for a direct comparison, see also Actis-Grosso and Ricciardelli, 2017; Ciardo et al., 2018).

Statistical analyses were conducted via repeated-measure analysis of variance (ANOVA) or via Friedman ANOVA when the data did not meet the assumption of normality (tested with the Shapiro-Wilk test). When significant ANOVA effects emerged, we further explored the results with paired samples *t*-tests and the family-wise error rate was controlled with the Holm–Bonferroni method (Holm, 1979). When data violated the assumption of normality, Wilcoxon signed-rank tests were used instead of paired *t*-tests. Effect sizes were computed by calculating the appropriate index out of the following: the partial eta squared index ($\eta_{p}^{2}$) for standard ANOVA, or the Kendall’s *W* (Sheskin, 2003) for Friedman ANOVA, or the *r*^2^ in Wilcoxon signed-rank tests for pairwise comparisons. Statistical analyses were implemented in IBM SPSS Statistics version 22.

#### Analysis of Distractor-Absent Trials

**FIGURE 2:**
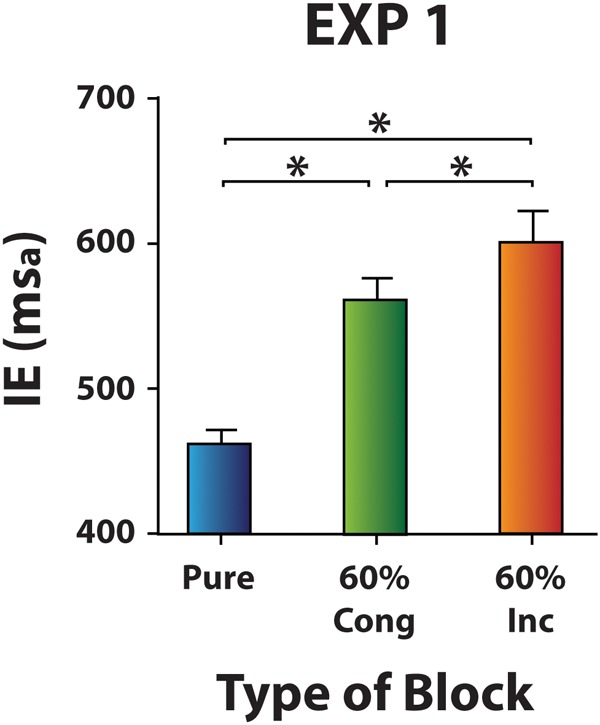
Distractor-absent trials in Pure and Mixed blocks (Experiment 1). Mean IE scores for distraction-absent trials in different blocks. Error bars represent the standard error of the mean. Asterisks mark significantly different mean/s.

Mean IEs on distractor-absent trials of Pure and Mixed blocks entered a one-way ANOVA factoring Block (Pure, 60% Cong, 60% Inc) and a significant effect was observed [χ^2^(2) = 33.6, *p* < 0.001, *W* = 0.84]. Responses on distractor-absent trials were faster in Pure blocks (mean IE ± SD = 462 ± 39 ms_a_) compared both to 60% Cong blocks (mean IE ± SD = 561 ± 59 ms_a_) and to 60% Inc blocks (mean IE ± SD = 601 ± 87 ms_a_) (*z* = 3.92, *p* < 0.001, *r*^2^ = 0.38 for each comparison). It was found that distractor-absent trials were faster in 60% Cong blocks than in 60% Inc blocks (*z* = 3.01, *p* = 0.003, *r*^2^ = 0.23; **Figure 2**). These results indicate distractor-absent trials were overall slower in Mixed than in Pure blocks and the magnitude of the Mixed blocks cost was larger in 60% Inc (vs. 60% Cong) blocks, possibly reflecting likelihood of conflict.

#### Sequential Effects on Distractor-Absent Trials

**FIGURE 3:**
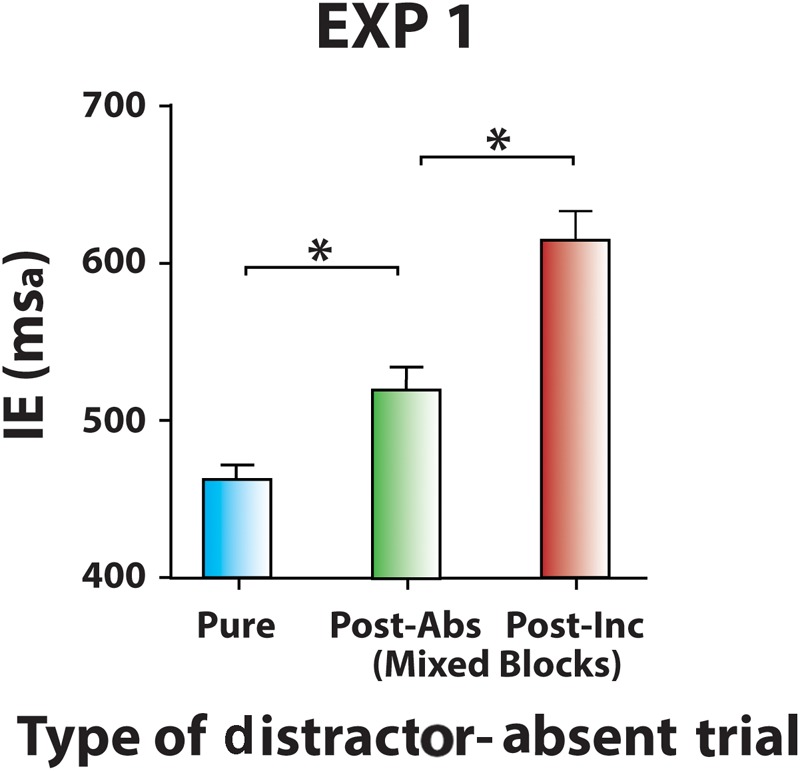
Sequential effects on distractor-absent trials (Experiment 1). Mean IEs on distractor-absent trials in Pure block, in Mixed blocks following distractor-absent trials and in Mixed blocks following incongruent distractor trials. Data of Mixed blocks come from collapsing 60% Cong block and 60% Inc block data. Error bars represent the standard error. Asterisks mark significantly different mean/s.

Inverse efficiencies for distractor-absent trials were sorted according to the Type of Preceding Trial and analyzed with a two-way ANOVA factoring Block (60% Cong block vs. 60% Inc block) and Type of Preceding Trial (distractor-absent vs. congruent distractor vs. incongruent distractor). The main effect of Block and the main effect of Type of Preceding Trial were both significant [*F*(1,19) = 6.82, *p* = 0.017, $\eta_{p}^{2}$ = 0.26, and *F*(2,38) = 44.0, *p* < 0.001, $\eta_{p}^{2}$ = 0.70, respectively], while their interaction was not [*F*(2,38) = 1.61, *p* = 0.40]. Data for each type of trial were then collapsed across types of Mixed blocks. Distractor-absent trials following another distractor-absent trial (mean IE ± SD = 519 ± 62 ms_a_) were faster than those following an incongruent distractor trial (mean IE ± SD = 614 ± 84 ms_a_) [*t*(19) = 8.84, *p* < 0.001, $\eta_{p}^{2}$ = 0.80]. Moreover, distractor-absent trials of Pure blocks (mean IE ± SD = 462 ± 39 ms_a_) were faster than distractor-absent trials of Mixed blocks following a distractor-absent trial [*t*(19) = 7.45, *p* < 0.001, $\eta_{p}^{2}$ = 0.75]. This pattern of results (**Figure 3**) indicates that the distractor-expectation cost was observed even at the net of any reactive conflict adaptation effect carrying-over from the previous trial (**Table 1**).

**Table 1:** Congruency effect as a function of previous trial congruency and type of Mixed block.

		Congruency effect	
		Type of Mixed block	
		60% Cong	60% Inc
**Previous trial**	Congruent	482 ± 246 ms_a_	269 ± 237 ms_a_
	Incongruent	133 ± 246 ms_a_	–137 ± 230 ms_a_
**Gratton effect**		349 ± 228 ms_a_	406 ± 269 ms_a_

*Values in the first and second rows represent the mean congruency effect ± standard deviation. Values in the third row correspond to the mean Gratton effect ± standard deviation.*

#### Congruency Effects in Mixed Blocks

As before, IE scores were entered a two-way ANOVA with factors Block (60% Cong vs. 60% Inc) and Type of Trial (distractor-absent vs. congruent distractor vs. incongruent distractor). We found a marginally significant main effect of Block [*F*(1,19) = 4.4, *p* = 0.050, $\eta_{p}^{2}$ = 0.19], a significant main effect of Type of Trial [*F*(1.2,22.5) = 44.43, *p* < 0.001, $\eta_{p}^{2}$ = 0.70] and a significant interaction [*F*(1.1,21.4) = 54.6, *p* < 0.001, $\eta_{p}^{2}$ = 0.74]. These effects were then explored separately for the 60% Cong and 60% Inc blocks with a one-way ANOVA (each) factoring Type of Trial (distractor-absent vs. congruent distractor vs. incongruent distractor).

**FIGURE 4:**
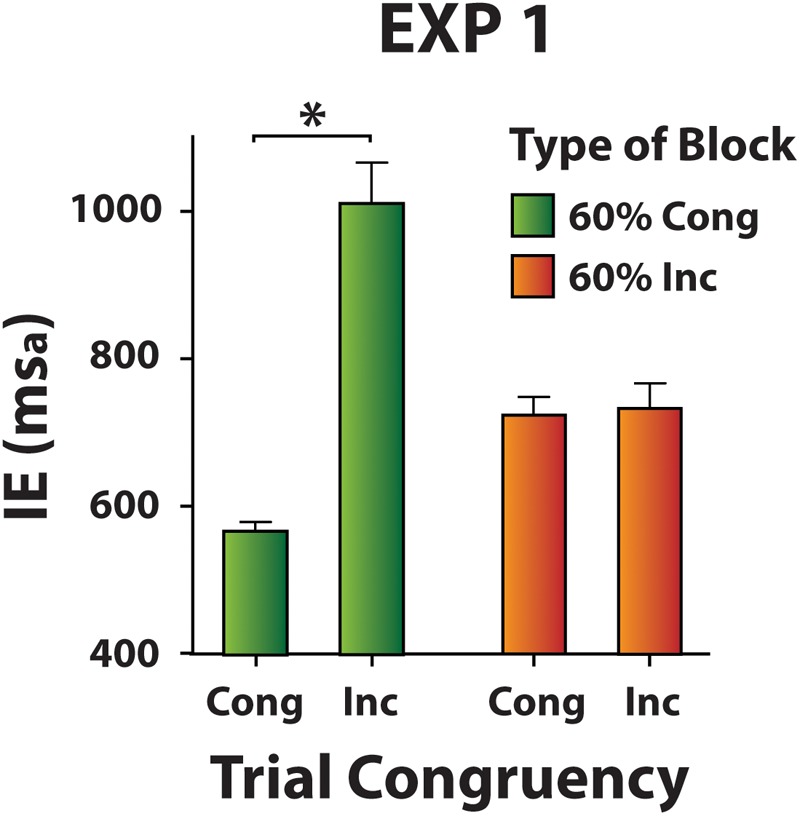
Distractor-present trials in Mixed blocks (Experiment 1). Mean IE values show a positive congruency effect in the 60% Cong blocks and no congruency effect in the 60% Inc blocks. Asterisks mark significantly different mean/s.

In the 60% Cong block, this analysis revealed a significant effect [χ^2^(2) = 30.4, *p* < 0.001, *W* = 0.76]. Incongruent distractor trials (mean IE ± SD = 1013 ± 239 ms_a_) were slower compared both to distractor-absent trials (mean IE ± SD = 562 ± 59 ms_a_) and to congruent distractor trials (mean IE ± SD = 568 ± 50 ms_a_) (*z* = 3.92, *p* < 0.001, *r*^2^ = 0.38 in both comparisons). These results attest to a positive congruency effect in 60% Cong blocks (**Figure 4**).

In 60% Inc blocks, the effect of Type of Trial was significant [χ^2^(2) = 24.4, *p* < 0.001, *W* = 0.61]. Distractor-absent trials (mean IE ± SD = 601 ± 87 ms_a_) were faster compared both to congruent (mean IE ± SD = 725 ± 105 ms_a_) and to incongruent (mean IE ± SD = 734 ± 152 ms_a_) distractor trials (*z* = 3.73, *p* < 0.001, *r*^2^ = 0.35, and *z* = 3.88, *p* < 0.001, *r*^2^ = 0.38, respectively). Mean IEs on congruent and on incongruent distractor trials were not significantly different (*z* = 0.37, *p* = 0.71), indicating the absence of any congruency effect in 60% Inc blocks. Furthermore, IEs within the same type of distractor-present trials were compared between Mixed blocks. Congruent and incongruent distractor trials were faster in the 60% Cong and in the 60% Inc blocks, respectively (i.e., in those blocks where the specific type of trial, congruent or incongruent, was more frequent) [*t*(19) = 7.32, *p* < 0.001, $\eta_{p}^{2}$ = 0.74, and *t*(19) = 6.65, *p* < 0.001, $\eta_{p}^{2}$ = 0.70, respectively (**Figure 4**)].

#### Gratton Effect

In order to investigate the Gratton effect (Gratton et al., 1992), a 2 × 2 ANOVA was run on congruency effect values (i.e., the IE-differences on incongruent minus congruent trials), with Block (60% Cong vs. 60% Inc blocks) and Previous Trial Congruency (congruent vs. incongruent) as factors. The main effect of Block was significant [*F*(1,19) = 25.6, *p* < 0.001, $\eta_{p}^{2}$ = 0.57], the main effect of Type of Preceding Trial was significant [*F*(1,19) = 55.7, *p* < 0.001, $\eta_{p}^{2}$ = 0.75], and their interaction was not significant [*F*(1,19) = 1.47, *p* = 0.24]. Interestingly, conflict in the preceding trial reduced the magnitude of the congruency effect (Gratton effect), likely due to adaptation to conflict, and did so independently of the probability of conflict at the block level (**Table 1**).

## Experiment 2

### Materials and Methods

#### Participants

Fifteen new participants took part in Experiment 2 (mean age 23.1, range 21–25, 11 females, 12 right-handed). All participants were recruited as before, had normal or corrected-to-normal vision, were unaware of the purpose of the research and the experimental procedure, and gave their written informed consent before testing.

#### Apparatus, Materials, and Procedure

**FIGURE 5:**
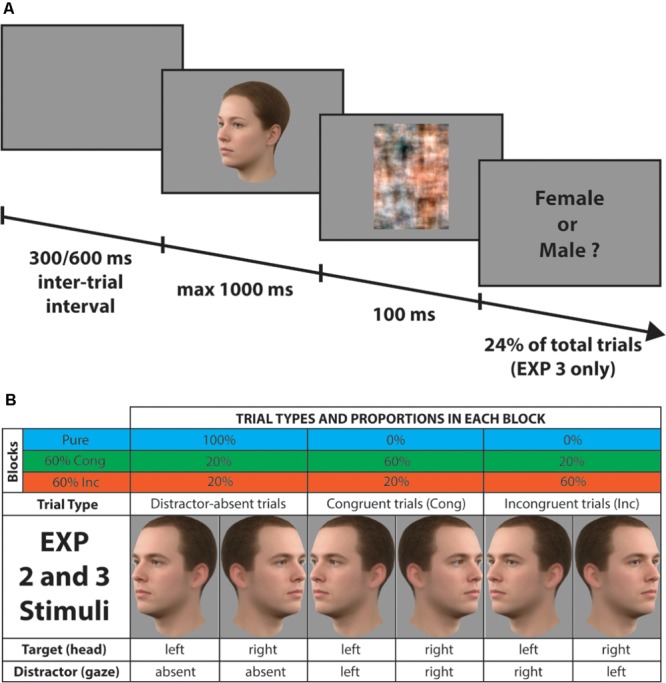
Examples of stimuli, trial types, and procedure (Experiments 2 and 3). **(A)** Example of a single trial. After 300 to 600 ms, a face appeared for 1000 ms at most and the participant responded according to the gaze direction. A mask followed the face for 100 ms. In Experiment 3, a question about the gender of the face was randomly presented after the mask in 24% of the total trials. **(B)** Table of the proportion of the different trial types in each block. In the lower part of the table, the pictures are the complete male set of stimuli with the description of gaze and head orientation beneath them. The female set is not shown here for brevity, but see **Figure 1**.

The design was similar to that of Experiment 1. The stimuli were the same Caucasian faces (male/female) as those used in Experiment 1 with different gaze orientations (straight/left/right) and different head orientations (tilted 45° left/right). The combination between these gaze and head orientations yielded a total of 12 unique face stimuli (**Figure 5**).

Each trial started with the presentation of a face stimulus (subtending a visual angle of 14.5° vertically by 10° horizontally) on a uniform gray background. Participants were instructed to indicate the head orientation (“target” dimension; left or right) as fast and as accurately as possible, while ignoring gaze orientation (“distractor” dimension; left, straight, or right). The face stimulus stayed on-screen until either participant’s response or for 1000 ms (whichever occurred first), and was immediately followed by a visual mask (100 ms). The inter-trial interval was jittered between 300 and 600 ms. Three types of trial were used: (1) distractor-absent trials, with averted gaze (i.e., the gaze was always straight); (2) congruent distractor trials, with the gaze averted in the same direction as the head orientation (either both left or both right); and (3) incongruent distractor trials, with the gaze averted in the opposite direction as the head orientation (either gaze left and head right, or vice-versa).

In order to investigate the functioning of proactive mechanisms for controlling the conflict emerging when gaze direction was task-irrelevant, we used the same distraction context manipulation as that used in Experiment 1. Specifically, two types of blocks were employed: Pure blocks, in which distractor-absent stimuli were presented on 100% of trials, and two Mixed blocks, a 60% Congruent block (i.e., consisting of 60% congruent distractor trials, 20% incongruent distractor trials, and 20% distractor-absent trials) and a 60% Incongruent block (i.e., consisting of 60% incongruent distractor trials, 20% congruent distractor trials, and 20% distractor-absent trials). Every block was preceded by a cue on the screen informing participants about the type of upcoming block (Pure, 60% Cong, 60% Inc). Prior to the beginning of the experiment, written instructions and examples of stimuli were shown on the screen and participants performed 30 practice trials. There were 960 trials in total divided in 15 blocks (5 blocks of each type, presented in a counterbalanced sequence) and had an average duration of about 25 minutes.

### Results

Reaction time and response accuracy were measured. RTs were filtered to eliminate outliers, defined as those trials on which RT was either below 200 ms or above the mean plus three standard deviations computed in log values (Ratcliff, 1993). IE scores were calculated as in Experiment 1 and all statistical analyses were performed as before.

#### Analysis of Distractor-Absent Trials

**FIGURE 6:**
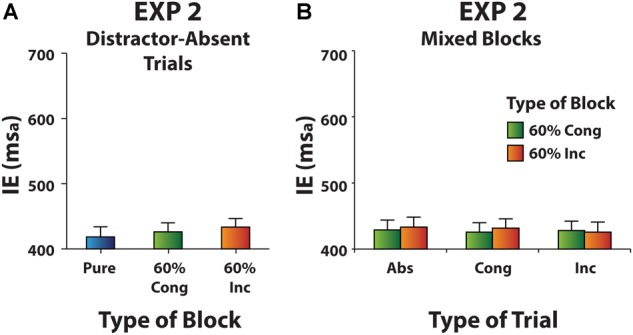
**(A)** Experiment 2: Mean IE scores for distractor-absent trials in Pure and Mixed blocks. **(B)** Experiment 2: Mean IE scores in Mixed blocks as a function of Type of Trial (distractor-absent, congruent distractor, and incongruent distractor), separately for 60% Cong block and 60% Inc block data. Error bars represent the standard error of the means across participants.

Inverse efficiency scores measured in the distractor-absent trials were submitted to a one-way ANOVA with Block as a three-level factor (Pure, 60% Cong, 60% Inc). We did not find a significant effect of Block [χ^2^(2) = 2.5, *p* = 0.282, *W* = 0.08], suggesting the absence of a filtering cost (**Figure 6A**).

#### Congruency Effects in Mixed Blocks

IE scores measured in Mixed blocks entered a two-way ANOVA with Type of Block (60% Cong vs. 60% Inc) and Type of Trial (distractor-absent vs. congruent distractor vs. incongruent distractor) as within-subjects factors. Neither the main effects [Type of Block: χ^2^(1) = 1.7, *p* = 0.20, *W* = 0.11; Type of Trial: χ^2^(2) = 0.40, *p* = 0.82, *W* = 0.01] nor their interaction [χ^2^(5) = 2.54, *p* = 0.77, *W* = 0.03] were significant, indicating that gaze direction information did not provide any distraction and could be easily filtered out (**Figure 6B**).

## Experiment 3

The present experiment was aimed to test whether increasing the task load would increase the distracting power of gaze direction when task irrelevant as it was in Experiment 2.

### Materials and Methods

#### Participants

Twenty-two new participants took part in Experiment 3 (mean age 22.2, range 19–25, 12 females, 21 right-handed). They were all recruited as before, had normal or corrected-to-normal vision, were unaware of the purpose of the research and the experimental procedure, and gave their written informed consent before testing.

#### Apparatus, Materials, and Procedure

They were the same as in Experiment 2 except that now in order to increase the task load, a question about the gender of the face stimulus was randomly presented on screen after the mask for only a subset of the total trials (24%) (**Figure 5**). The gender discrimination task was chosen on the base that gender, different from gaze direction and head orientation, is an invariant facial property that interacts with changeable facial aspects (e.g., Karnadewi and Lipp, 2011). Therefore, we reasoned that both the changeable and invariant proprieties of the face (i.e., head orientation and gender) in the present experiment had to be processed (although only in 24% of trials) thus increasing the task load compared to Experiments 1 and 2 where only changeable aspects of the face needed to be taken into account.

### Results

All statistical analyses were performed as before. In addition, for a comparison of IE scores between Experiments 2 and 3, we conducted a Mann–Whitney test in distractor-absent trials and Mixed blocks, separately. Eta-squared for Mann–Whitney test was defined as *z*^2^/*N*-1 (Hatch and Lazaraton, 1991).

#### Analysis of Distractor-Absent Trials

**FIGURE 7:**
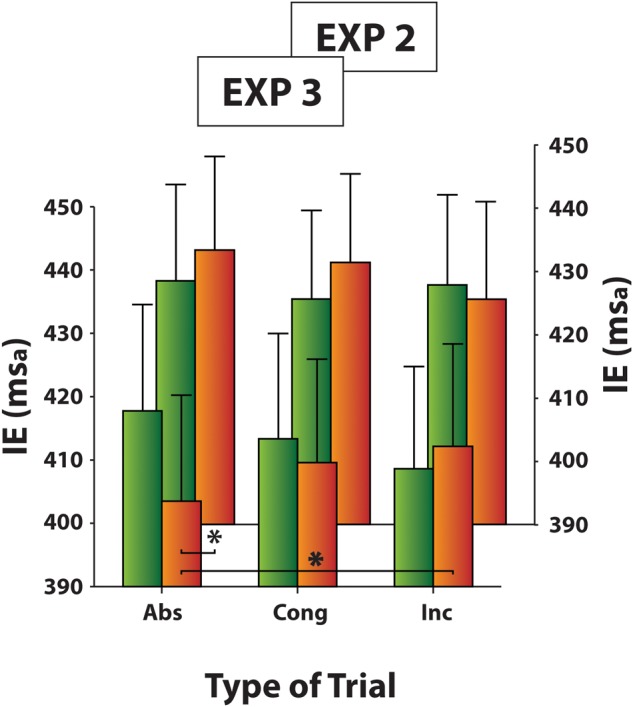
Experiment 3: Mean IE scores of distractor-absent trials in Pure and Mixed blocks (60% Cong block and 60% Inc block). Results of Experiment 2 are also plotted on background for a comparison. Error bars represent the standard error of the means across participants. Asterisks mark significantly different mean/s.

As in the previous experiment, IE scores measured in the distractor-absent trials were submitted to a one-way ANOVA with Type of Block as a three-level factor (Pure, 60% Cong, 60% Inc). The analysis revealed that the effect of Type of Block was significant [χ^2^(2) = 6.8, *p* < 0.04, *W* = 0.15; **Figure 7**]. Responses to distractor-absent trials were slower in 60% Cong block (mean IE ± SD: 412 ± 74 ms_a_) compared both to Pure block (mean IE ± SD: 404 ± 77 ms_a_; *z* = -2.22, *p* < 0.03, *r*^2^ = 0.94) and 60% Inc block (mean IE ± SD: 403 ± 76 ms_a_; *z* = -1.96, *p* = 0.05, *r*^2^ = 0.92) which did not differ from each other (*z* = -0.21, *p* = 0.83). The comparison between Experiments 2 and 3 approached significance for the 60% Inc block (*U* = 104.00, *p* = 0.05, $\eta_{p}^{2}$ = 0.10), suggesting the presence of a top-down/strategic cost only. This is likely because, since the participants were expecting to perform a second task, they paid more attention to the face gender thus improving their performance compared to Experiment 2, especially in distractor-absent trials.

#### Sequential Effects on Distractor-Absent Trials

**FIGURE 8:**
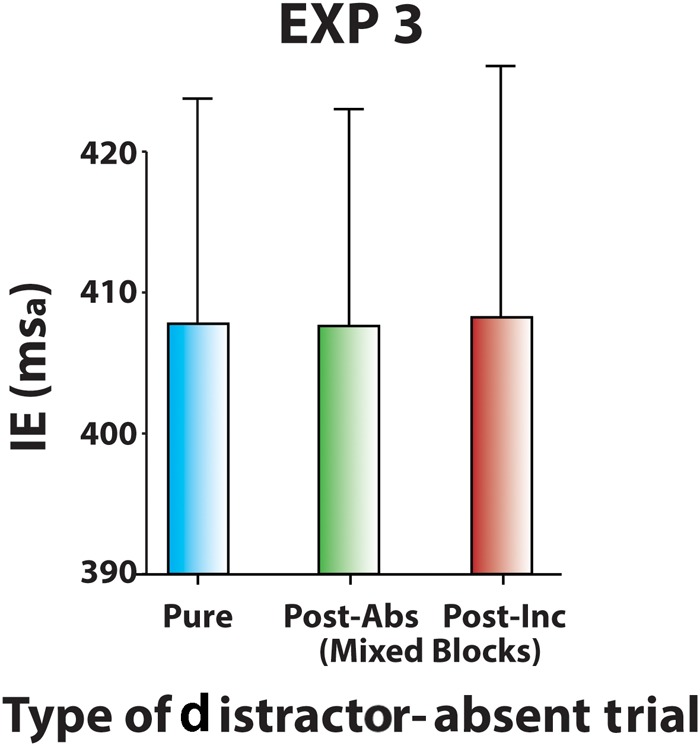
Experiment 3: Sequential effects on mean IEs of distractor-absent trials in Pure block, in Mixed blocks following distractor-absent trials, and in Mixed blocks following incongruent distractor trials. Data of Mixed blocks were obtained by collapsing 60% Cong block and 60% Inc block data. Error bars represent the standard error of the means across participants.

To test the possible effect of conflict in the preceding trial on responses, distractor-absent trials of Mixed blocks were sorted according to the Type of Preceding Trial and analyzed with a two-way ANOVA factoring Type of Block (60% Cong block vs. 60% Inc block) and Type of Preceding Trial (distractor-absent vs. congruent distractor vs. incongruent distractor). Neither the main effects [Type of Block: χ^2^(1) = 2.9, *p* = 0.09, *W* = 0.13; Type of Preceding Trial: χ^2^(2) = 1.1, *p* = 0.58, *W* = 0.02] nor their interaction [χ^2^(5) = 4.9, *p* = 0.43, *W* = 0.04] were significant, indicating none reactive conflict adaptation effect carrying-over from the previous trial but a proactive adaptation only (**Figure 8**).

#### Congruency Effects in Mixed Blocks

Again IE scores measured in Mixed blocks were submitted to a two-way ANOVA with Type of Block (60% Cong vs. 60% Inc) and Type of Trial (distractor-absent vs. congruent distractor vs. incongruent distractor) as within-subjects factors. The main effect of Type of Block [χ^2^(1) = 6.5, *p* < 0.02, *W* = 0.30] and the interaction between Type of Block and Type of Trial [χ^2^(5) = 12.0, *p* < 0.04, *W* = 0.11] were significant. Moreover, the comparison between Experiments 2 and 3 approached significance for distractor-absent trials (*U* = 104.00, *p* = 0.05, $\eta_{p}^{2}$ = 0.10), suggesting an overall increase of attention (top-down/strategic control)when a second task (the discrimination of face gender) needs to be performed. Each type of Mixed block was then explored with a one-way ANOVA factoring Type of Trial (distractor-absent vs. congruent distractor vs. incongruent distractor).

**FIGURE 9:**
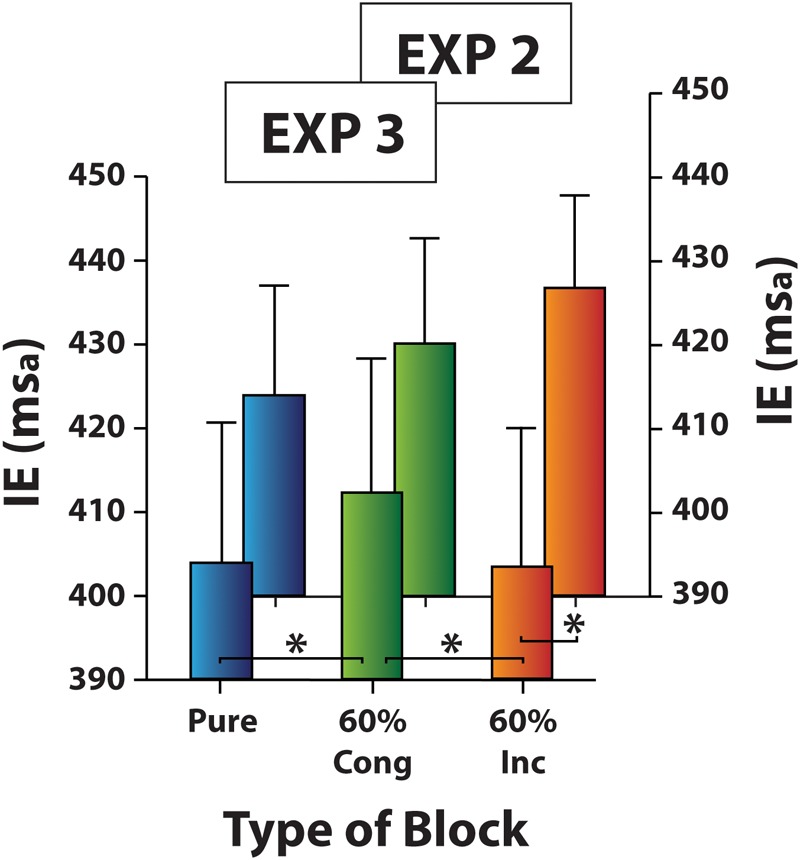
Experiment 3: Mean IE scores in Mixed blocks as a function of Type of Trial (distractor-absent, congruent distractor, and incongruent distractor), separately for 60% Cong block and 60% Inc block data. Results of Experiment 2 are also plotted on background for a comparison. Error bars represent the standard error of the means across participants. Asterisks mark significantly different mean/s.

Within 60% Cong blocks, an effect of blocks was found to approach significance [χ^2^(2) = 5.8, *p* = 0.06, *W* = 0.13], indicating slower reaction times in distractor-absent trials (mean IE ± SD: 418 ± 77 ms_a_) relative to both congruent (mean IE ± SD: 413 ± 76 ms_a_; *z* = -2.00, *p* = 0.05, *r*^2^ = 0.97) and incongruent distractor trials (mean IE ± SD: 409 ± 73 ms_a_; *z* = -2.16, *p* < 0.04, *r*^2^ = 0.94), which did not differ from each other (*z* = -1.18, *p* = 0.24; **Figure 9**).

Within 60% Inc blocks, an effect of blocks was found to approach significance [χ^2^(2) = 5.8, *p* = 0.05, *W* = 0.13], indicating faster reaction times in distractor-absent trials (mean IE ± SD: 403 ± 76 ms_a_) relative to incongruent distractor trials (mean IE ± SD: 412 ± 74 ms_a_; *z* = -1.96, *p* = 0.05, *r*^2^ = 0.92; all the other *p*s were >0.12, **Figure 9**).

#### Gratton Effect

In order to investigate the Gratton effect, a 2 × 2 ANOVA was run on congruency effect values (i.e., the IE-differences on incongruent minus congruent trials), with Type of Block (60% Cong vs. 60% Inc blocks) and Previous Trial Congruency (congruent vs. incongruent) as factors. Neither the main effects [Type of Block: *F*(1,21) = 0.26, *p* = 0.61, $\eta_{p}^{2}$ = 0.01; Previous Trial Congruency: *F*(1,21) = 0.89, *p* = 0.36, $\eta_{p}^{2}$ = 0.04] nor their interaction [*F*(1,21) = 0.82, *p* = 0.38, $\eta_{p}^{2}$ = 0.04] were significant, indicating that conflict in a preceding trial had no effect on the following trial.

## Discussion

In the present study, we tested whether proactive control processes for filtering-out irrelevant stimulus information (e.g., Marini et al., 2013) were recruited in a social attention task where participants made a speeded directional judgment based on either head orientation or gaze direction cues in contexts with varying probability of conflict between the two cues. In particular, we were interested in studying whether the weights attributed to directional information coming from the head and the eyes during the integration of multiple and conflicting cues could be adjusted based on the prior knowledge of different probabilities of conflict, thus affecting the head-gaze congruency effect (RT in incongruent minus congruent trials). Proportion of conflict (incongruent trials) within Mixed blocks (Experiments 1–3) and cognitive load (Experiment 3) were taken into account as variables that could potentially modulate the congruency effect. If head orientation and gaze direction could be filtered out when task irrelevant, then a distraction-filtering cost (i.e., slower responses and larger IE score on distractor-absent trials of Mixed blocks relative to the same trials of Pure blocks) should be found both for head and gaze cues. In addition, the congruency effect should be modulated both proactively and reactively by conflict probability. Finding these results when both the head and the gaze are task irrelevant would suggest similar weights for these directional cues in the integration process. By contrast, the absence of a distraction-filtering cost and thus the lack of proactive and reactive modulation when gaze direction or head orientation is irrelevant, would indicate a difference in the weights of head and gaze cues.

In Experiment 1, where head orientation was irrelevant, we found that the Mixed blocks cost was modulated by the frequency of conflict and the presence or absence of conflict in the previous trial. Specifically, distractor-absent trials in Mixed blocks following an incongruent distractor trial were slower than those following a distractor-absent trial. This is the evidence of a reactive trial-to-trial adjustment triggered by the conflict occurrence. However, distractor-absent trials following a distractor-absent trial in Mixed blocks were slower than distractor-absent trials following a distractor-absent trial in Pure blocks suggesting that the observed slowing-down is not fully accounted for trial-to-trial adjustments, but also involves a proactive component. This adaptation may be guided by previous knowledge of the probability of conflict, and/or by the contingent trial history of proportion congruency.

The congruency effect was present in 60% Cong blocks whereas it was absent in 60% Inc blocks. Interestingly then, in those Mixed blocks where a specific type of distractor-present trial (congruent or incongruent) was relatively frequent, responses were also faster than for the relatively infrequent trial type (see **Figure 4**). This indicates that participants have benefitted from prior knowledge about the probability of conflict at the block level. This suggests that the control of proactive distraction-filtering at the block-level and the reactive trial-to-trial adjustments to conflicting distraction may be controlled by different dynamics. One possibility is that a central monitoring system, whenever conflict occurs, triggers reactively a top-down control mechanism that enhances distraction filtering in the next trial, which results in the observed reduction of interference. Some authors (Nieuwenhuis et al., 2006; Lamers and Roelofs, 2011) did not consider the Gratton effect explainable by the conflict-monitoring hypothesis (Botvinick et al., 2001) and suggested that simple stimulus or response repetition may account for this effect. In Experiment 1, when a given type of trial was repeated, the Gratton effect was different based on the block-level probability of conflict, which does not seem to be compatible with the stimulus or response repetition account of the Gratton effect. Instead, because the conflict-probability context at the block-level modulated the magnitude of the Gratton effect, our findings concur with the idea that a general conflict-monitoring system may control trial-to-trial the magnitude of the congruency effect in the current paradigm.

On the contrary, as expected, in Experiment 2 where gaze direction was the distracting information and head orientation was the target dimension, no filtering cost was found and no congruency effect emerged either. However, in Experiment 3 when the cognitive load of the head orientation discrimination task was increased by asking participants to perform a gender discrimination task as well as a head discrimination one (in 24% of the trials), the Mixed block cost emerged again as in Experiment 1. Specifically, in Experiment 3 we found slower responses to distractor-absent trials in 60% Cong blocks than in Pure blocks and in 60% Inc blocks, whereas responses to distractor-absent trials between the latter two block types were not different from each other. Moreover, unlike in Experiment 1, no reactive conflict adaptation effects carried over from the previous trial emerged. Finally, no significant congruency effects were found in 60% Cong blocks or in 60% Inc blocks, and no Gratton effect emerged either.

Our findings in Experiments 1 and 3 speak in favor of the recruitment a proactive control mechanism that is at play also when we process conflicting directional social cues coming from the face, such as head orientation and gaze direction. In particular, similar to what has been already reported for non-social stimuli (Marini et al., 2013), this distraction-filtering mechanism is sensitive to *contextual factors* and intervenes to *filter-out one source of information* (e.g., eye cues or head cues) *from the cue integration process* when the observer has prior knowledge that a source of information is irrelevant within a given face-processing context. Specifically, knowing in advance the conflict probability modulates a proactive control mechanism whose function is to maintain active the current task goals (Braver, 2012), thus allowing the observer to perform a directional discrimination judgment task of either the gaze or the head. Interestingly, however, our results clearly demonstrate a qualitative different pattern of results between the two tasks, suggesting that head and gaze cues are processed together and when one of the two is task irrelevant the weight of the interference is not the same. In particular, in a speeded judgment task as used in the present study, the interference of head orientation on gaze direction judgments was stronger, as indicated both by the presence of the filtering distracting cost in Experiment 1 and its absence in Experiment 2. In parallel, a proactive distraction-filtering cost was also absent in Experiment 2, suggesting an inherently larger weight attributed to head orientation and a reduced distracting power of the gaze direction in this task. This is in line with the finding reported by Ricciardelli and Driver (2008) who found effects of the congruency between head orientation and gaze direction in a left/right gaze judgment task. As in the present study (Experiment 1), when a speeded judgment of gaze direction was required, faster RTs were found when the head and gaze directions deviated toward the same side (congruency effect; see also Langton, 2000), but only when the full face was visible (global processing). Therefore, under time pressure, the head orientation of the whole face becomes weighted more heavily, so that gaze deviations toward the same direction as the tilted head then become easier to judge rapidly. Indeed, our findings from Experiment 1 show that the distraction-filtering cost in blocks with rare conflict (60% Cong blocks) was comparably smaller than the one in blocks with frequent conflict (60% Inc blocks) where the inhibition of task-irrelevant information required was stronger (e.g., Ridderinkhof, 2002; Braver, 2012).

Moreover, the finding that the distraction-filtering cost when gaze direction is the distracting information, emerges only with higher cognitive load (Experiment 3) is in line with the idea that the cognitive load can reduce the efficiency of distraction-filtering by increasing both the distraction-filtering cost and the congruency costs (cf. Experiments 5 and 7 in Marini et al., 2013), particularly in the blocks with frequent distraction. However, the difference between Experiments 2 and 3 is only marginal, and increasing the cognitive load within subjects would have been a better manipulation. Nevertheless, the findings of the present study suggest that the effect of cognitive load is likely because, in order to perceive the gender of the face, the whole face (head and the eyes) needs to be processed and attended. In a speeded task, filtering-out distraction proactively was more effortful when this distracting information came from a local feature such as the gaze direction (vs. head orientation). This explanation would account for the fact that in Experiment 2 (low cognitive load) we did not found any the distraction-filtering cost and in Experiment 3 we found it only for distractor-absent trials in Mixed blocks with rare conflict. This is a new finding that extends previous evidence (e.g., Perrett et al., 1992; Sugase et al., 1999; Watanabe et al., 1999; Ricciardelli and Driver, 2008) of a hierarchical organization and processing of head and gaze cues. Taken together, the present and previous results suggest that the visual system may give different weights to head and gaze cues according to different contexts (i.e., the required speed of the judgment, the expectation of conflict and cognitive load). The hierarchy of cues may weight head direction cues more strongly when a speeded visual judgment is required (perhaps because head orientation is a global visual property of face and is extracted more rapidly than local features; e.g., Sugase et al., 1999). This would explain the pattern of engagement of a distraction-filtering mechanism and the presence of congruent effects observed in the current study.

Another aspect emerging from the present findings concerns the integration of different directional cues (head orientation and gaze direction) of the face that gives rise to the two biases (a repulsive effect and the attraction effect) in perceived gaze direction discussed in literature when eye and head orientation are conflicting (Moors et al., 2016). Since the present study and a previous study by Ricciardelli and Driver (2008) showed that the weight of these cues in the integration process could be modulated both by the frequency of conflict and by time constraints, these two biases may change as a function of both distraction expectation and speeded task requirement. Further research is needed to test this hypothesis. Moreover, a possible limitation of our study is that we only investigated the top-down modulation triggered by the instruction.

Overall, our study demonstrates that, during attention to social cues, proactive cognitive control mechanisms are modulated by the expectation of conflicting stimulus information, and that conflict adaptation mechanisms intervene flexibly in order to facilitate decisions that are most frequently required depending on the specific task context. Unlike previous results with non-social stimuli (Marini et al., 2013), here the reactive adaptation to conflict as a function of previous trial congruency (i.e., the Gratton effect) was not modulated by the distraction probability at the block level. This result may be specifically related to the processing of social direction cues, and suggests a weaker strategic top-down control when processing social and biological stimuli (see also Marino et al., 2015). In a similar vein, a recent study investigating the conflict between the spatial information conveyed by non-informative gaze and arrow direction and the target spatial position in cueing task reported that, unlike other conflict tasks (the Simon task), in the gaze and arrow cueing task the previous-trial congruence modulated only responses to the following congruent trials (Ciardo et al., 2018). The fact that the congruency of the preceding trial did not affect performance in the subsequent incongruent trials is in line with the idea of a failure of an inhibitory mechanism in suppressing the automatic orienting of attention triggered by gaze and over-learnt directional cues (such as arrows), even following a conflicting event.

In conclusion, the present study shows that filtering-out potentially conflicting head information is a more resource-demanding process than filtering out conflicting gaze direction and entails a cost likely related to proactive control mechanisms. Accordingly, this cost is larger when conflict occurs frequently (vs. rarely), but the opposite was true when gaze direction was irrelevant and the task cognitive load was increased.

Perceiving where someone else is attending relies on the integration of multiple social cues. This integration process can be modulated by proactive control mechanisms that are sensitive to the context (being the probability of encountering conflicting social cues within a given experimental block). These cognitive control mechanisms consist of proactive slowing-down due to the expectation of distraction and conflict adaptation. Together, they help the focusing on relevant cues (e.g., gaze) and away from irrelevant ones (e.g., head orientation).

## Author Contributions

AR, FM, and PR conceived the experiments. AR implemented the study and collected part of the data. AR and BM analyzed the data and drew the figures. All authors drafted the manuscript, provided critical revisions, and approved the final version of the manuscript.

## Conflict of Interest Statement

The authors declare that the research was conducted in the absence of any commercial or financial relationships that could be construed as a potential conflict of interest. The reviewer MD and the handling Editor declared their shared affiliation.

## Footnotes

1 The other bias is the towing or attraction effect, which indicates that perceived gaze direction falls in between head and eye orientation (e.g., Moors et al., 2016).
